# Real-World Impact of Remote Patient Management on Care Consumption and Care Time in Heart Failure: Retrospective Cohort Study

**DOI:** 10.2196/89187

**Published:** 2026-09-03

**Authors:** Wessel William Nieuwenhuys, Mayke Maria Christina Josephina van Leunen, Jeroen Albert Adrianus van de Pol, Frederique Jantine Hafkamp, Rudolph Ferdinand Spee, René Anton Tio, Mathias Funk, Hareld Marijn Clemens Kemps

**Affiliations:** 1 Industrial Design Department, Eindhoven University of Technology, Groene Loper 3, Eindhoven, North Brabant, 5612 AE, The Netherlands, 31 040 247 9111; 2 Eindhoven MedTech Innovation Centre (e/MTIC), Eindhoven, North Brabant, The Netherlands; 3 Nederlands Hart Netwerk, Eindhoven, North Brabant, The Netherlands; 4 Department of Cardiology, Máxima Medisch Centrum, Veldhoven, North Brabant, The Netherlands; 5 Department of Electrcial Engineering, Eindhoven University of Technology, Eindhoven, North Brabant, The Netherlands; 6 Department of Medical and Clinical Psychology, Tilburg University, Tilburg, North Brabant, The Netherlands; 7 Department of Cardiology, Catharina Ziekenhuis, Eindhoven, North Brabant, The Netherlands

**Keywords:** heart failure, cardiology, remote patient management, remote patient monitoring, retrospective study, telemedicine, workload, patient outcome assessment

## Abstract

**Background:**

Remote patient management (RPM) is a strategy to track daily health data in patients with heart failure (HF) to enable early detection and treatment of decompensation to prevent hospital readmissions. The implementation of RPM alters standard care and redistributes provider responsibilities. Although randomized controlled trials demonstrate positive clinical outcomes, the impact of implementing RPM on patients’ health care usage, as well as the care time and workload of health care providers in a real-world setting, remains unclear.

**Objective:**

The objective of this retrospective study is to explore the effects of implementing RPM for HF on patients’ cardiac care consumption and caregiver workload within a regional care network through the use of real-world data.

**Methods:**

Two hospitals in the same regional care network with aligned care pathways for usual care (UC) were compared: one with RPM (hospital 1) and one without (hospital 2). Between June 2021 and July 2022, patients with HF were included from a clinical registry with a 1-year follow-up. Cardiac health care consumption was quantified by the number and duration of consultations (in-person, phone, and RPM) with nurse practitioners specialized in HF care and cardiologists. HF-related readmissions were also collected. Three groups were analyzed: hospital 1 RPM, hospital 1 UC, and hospital 2 UC.

**Results:**

A total of 131 patients were included (hospital 1 RPM: 34, hospital 1 UC: 64, hospital 2 UC: 33). Consultation frequency and duration were significantly higher in the hospital 1 RPM cohort compared to the hospital 1 UC and the hospital 2 UC cohorts (frequency: mean 18.5, SD 9.3 vs mean 9.3, SD 6.0 and mean 11.6, SD 6.3; *P*<.001; duration: mean 233.2, SD 95.2 min vs mean 121.2, SD 47.9 min and mean 113.0, SD 66.0 min; *P*<.001), mainly due to increased number of consultations of nurse practitioners specialized in HF care (hospital 1 RPM: mean 15.3, SD 8.4 vs hospital 1 UC: mean 5.3, SD 2.4 and hospital 2 UC: mean 8.6, SD 6.1; *P*<.001). No significant differences were observed regarding cardiologist care time (*P*=.27), cardiologist number of consultations (*P*=.43), or HF-related readmissions (*P*=.47).

**Conclusions:**

In this real-world study, the implementation of RPM for patients with HF was associated with a higher nursing workload, with no observed differences in cardiologists’ consultations or patient outcomes. These findings highlight the need for optimization at organizational and technological levels and underline the importance of real-world evaluation of the implementation of innovations.

## Introduction

Heart failure (HF) is a chronic, progressive syndrome that results in poor quality of life, decreased functional capacity, and significant mortality. Globally, approximately 55 million people are living with HF, and its prevalence in Europe and North America is expected to double by 2040 [1]. The high prevalence, combined with escalating costs and a shortage of health care professionals, necessitates the implementation of innovative approaches to alleviate this growing burden on health care [2,3].

Remote patient management (RPM), which involves using technology to track patients’ health data remotely, has emerged as a promising solution. RPM enables timely interventions at the early onset of symptoms, potentially preventing hospital admissions, reducing unnecessary visits, decreasing mortality, and reducing the load on the health care system [4,5]. By shifting from in-person outpatient visits with medical specialists to nurse-led phone consultations, RPM has the potential to facilitate workload redistribution. Already, approximately 50% of Dutch hospitals have implemented RPM for patients with HF [6]. However, the benefits of RPM, often demonstrated in strongly protocolized randomized controlled trials (RCTs) with dedicated research teams, may not directly translate to real-world settings [7-9]. Furthermore, the implementation processes of RPM are often insufficiently described in existing studies, potentially missing systematic implementation challenges and shifts in workload arising from its integration into daily practice [10].

While RCTs can provide valuable insights into the efficacy of interventions, their controlled conditions often do not completely reflect the complexities of real-world practice. For example, during the implementation of a new intervention, health care providers frequently encounter obstacles that are not present in study contexts, such as limited resources in both time and manpower, lack of proper training, preexisting care pathways, and reluctance among staff to deviate from established routines [11-14]. To accurately reflect these obstacles and their impact on implementation efficacy in routine practice, real-world evidence should be considered to complement RCTs.

Real-world evaluations help determine whether an intervention achieves its intended outcomes in everyday care and provide essential feedback to refine implementation processes and facilitate smoother adoption of future innovations. In practice, however, such evaluations can be challenging. Researchers often encounter issues such as confounding effects of the start-up phase, incomplete data, and the lack of a control group receiving concurrent usual care (UC) under similar conditions [15,16]. These methodological and practical challenges inherent to real-world evaluations underscore the need for approaches that explicitly account for this complexity. Conducting such research within regional organizations or collaborative care networks can help address some of these challenges, as it enables comparisons across geographically proximate hospitals with similar patient populations and aligned care pathways, providing a strong basis for assessing the effects of new implementations.

This study aims to retrospectively compare HF care delivery at 2 hospitals within a regional care network in the Netherlands. Both hospitals adhere to the same established UC pathways; however, one also has an established RPM implementation (hospital 1 [H1]), whereas the other does not (hospital 2 [H2]). This study will describe differences in health care usage and workload distribution associated with RPM implementation using real-world data. To this end, we analyzed differences in the number and duration of different types of consultations and admissions during the first year following referral.

## Methods

### Research Setting

The Netherlands Heart Network (NHN) is a care network, fostering transmural communication between care providers, establishing standards for UC pathways, and setting innovation agendas with regard to heart care in the southeastern region of the Netherlands. Within the NHN, the shared goal is to continuously improve the value of care for patients with heart disease. To achieve this, cardiologists, nurse specialists, general practitioners, and practice nurses collaborate in an HF-focused caregiver-in-the-lead subnetwork. Within this group, transmural care standards are developed and innovation agendas are formulated to enable region-wide implementation [17,18].

Since 2018, one of the participating hospitals within the NHN has implemented an RPM care pathway for HF. Patients included in the care pathway used a web application to communicate HF-related vital signs daily to nurse practitioners. The goal of this implementation was to reduce rehospitalizations, mortality, and the amount of care time of both nurses and cardiologists.

#### Care Pathways

H1 (Máxima Medical Centre, Veldhoven, The Netherlands) offered RPM to patients with acute decompensated HF. To be eligible, patients needed to be willing and able to use the RPM system, and their care providers had to identify a potential clinical benefit for their participation. For patients not willing or able to participate in RPM, H1 also provided UC without RPM. H2 (Catharina Hospital, Eindhoven, The Netherlands) had not yet implemented RPM in HF care.

#### UC (H1 and H2)

Both hospitals are active participants in the regional care network, following the same established UC pathways. All patients were referred to the HF outpatient clinic after recent hospitalization or a new HF diagnosis and required medication up-titration to optimal medical therapy (OMT). UC outpatient follow-up included a nurse practitioner specialized in HF care in-person visit at week 2 and a cardiologist in-person visit at week 6, and then every 3 months (in-person or by phone). Additional appointments were scheduled as needed, such as for OMT up-titration or suspected HF deterioration.

#### RPM (H1)

In H1, clinical nurses and cardiologists screened all patients with chronic HF admitted for acute decompensated HF for RPM participation during their initial outpatient visit. If the care provider judged that there was potential clinical benefit from RPM participation, the process of remote monitoring and required patient actions were explained. To be eligible for RPM, patients needed proficiency in speaking and reading Dutch, as the web application used (MiBida BV [19]) was only available in Dutch, and they had to be willing and able to report daily measurements digitally.

Enrolled patients reported blood pressure, heart rate, and body weight and answered 4 HF-related symptom questions (eg, “Do you experience shortness of breath?”) daily. The system generated alerts based on personalized thresholds, prompting patients to contact a nurse practitioner specialized in HF care during set hours (urgent issues: 24/7). Patients were instructed to contact the nurse practitioner specialized in HF care by telephone between defined hours when an alert was generated. For urgent matters, medical professionals were available 24/7. Furthermore, a nurse practitioner specialized in HF care reviewed data, managed alerts, contacted patients, consulted cardiologists, and adjusted medication as needed. This RPM model, combining 3 vital parameters and a symptom questionnaire, aligns with the most commonly described approach in the literature [20]. Outpatient follow-up included a nurse practitioner specialized in HF care in-person visit at week 2 and a cardiologist in-person visit at week 6 (after hospital discharge), and then every 6 months (in-person or by phone). The frequency of visits to the cardiologist was reduced to annual visits for patients who remained stable for more than a year. Additional appointments were scheduled as needed, such as for OMT up-titration or suspected HF deterioration.

### Study Design and Participation

This multicenter retrospective cohort study evaluated adults with chronic HF who were newly referred to the HF outpatient clinic. Eligible patients were retrospectively identified from the HF dataset within the Netherlands Heart Registration (NHR) clinical data registry, a national quality registry with the aim of monitoring and improving the quality of cardiac care [21]. In the Netherlands, it is standard practice that data for patients with HF are registered in the HF dataset during the patient’s first outpatient visit with a nurse practitioner specialized in HF care and are subsequently followed up for up to 1 year. Eligible patients met the following inclusion criteria: diagnosis of chronic HF (regardless of etiology or left ventricular ejection fraction), aged ≥18 years, and a new referral to the HF outpatient clinic at H1 or H2 between June 2021 and July 2022. No formal sample size calculation for this study was performed, as the study aimed to assess the real-world implementation of remote patient monitoring using routinely collected clinical data. To reach the largest possible study population, all patients in the dataset who met the inclusion criteria were included.

**Figure 1.**
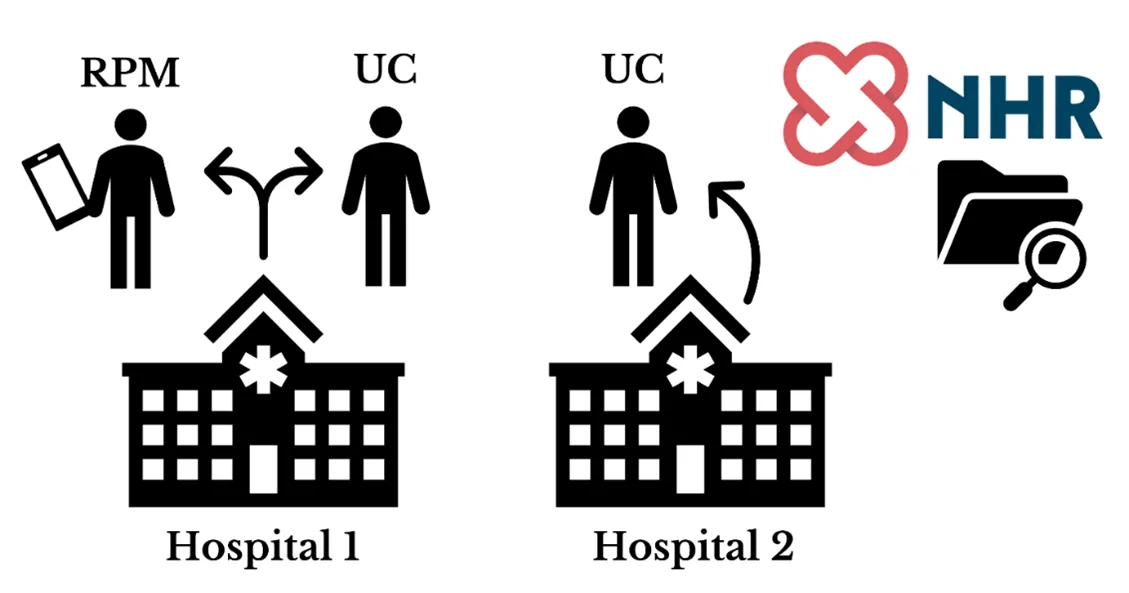
Visualization of the methods of how the 3 cohorts were formed. From hospital 1, patients were categorized into 2 cohorts based on their care pathway. Hospital 2 only provided usual care (UC) and thus provided only one cohort. Data for all patients were retrospectively obtained from the heart failure (HF) dataset within the Netherlands Heart Registration (NHR) clinical data registry and enhanced with a search through both hospital electronic health records. RPM: Remote Patient Management.

Enrolled patients were categorized into 3 cohorts, namely H1-RPM, H1-UC, or H2-UC, based on their care pathway, as shown in Figure 1. This allowed comparison both between hospitals and between care pathways within the same hospitals.

The reporting of this study followed the STROBE (Strengthening the Reporting of Observational Studies in Epidemiology) guidelines (Checklist 1) [22].

### Data Collection and Outcomes

The primary outcome was health care usage among patients with HF during the first year after inclusion, comparing RPM users and nonusers. This was assessed by retrospectively mapping hospital admissions, outpatient cardiology visits, other cardiology consultations, and emergency room visits. By categorizing these types of health care usage by care provider role and type of consultation, resulting shifts in workload distribution could be evaluated.

The NHR HF database provided patient demographic data. Health care usage was obtained from the hospitals’ electronic patient files and was quantified in terms of frequency and duration. Usage was categorized into consultations and hospitalizations. Consultations were further subdivided based on care provider (cardiologist and nurse practitioner specialized in HF care) and type (in-person or phone), with RPM consultations managed exclusively by nurse practitioners specialized in HF care. RPM consultations were defined as consultations related to RPM or initiated by an RPM alert and were distinctly marked as such in the dataset to differentiate them from UC consultations. In the electronic patient files, consultation times were estimated based on standardized durations assigned to specific consultation types (eg, new patient consultations, follow-up appointments, and home visits), rather than individually recorded start and end times. Only HF-related hospitalizations were included, with lengths of stay measured in days.

### Statistical Analysis

Demographic and clinical characteristics were summarized as frequencies (categorical variables) or means (SDs; continuous variables). Group differences were tested using chi-square tests (categorical) and one-way ANOVAs (continuous), with Tukey least significant difference for post hoc comparisons. Statistical analyses were conducted in SPSS Statistics for Windows (version 28.0; IBM Corp), with significance considered at *P*<.05.

### Ethical Considerations

After reviewing the study protocol, the Medical Ethics Committee of Máxima Medical Centre (the Netherlands) determined that the study, being a retrospective analysis, did not fall within the scope of the Medical Research Involving Human Subjects Act (Wet Medisch-Wetenschappelijk Onderzoek met Mensen) and therefore did not require further approval before commencement (METC-code: 2023-MMC-070). Due to the nature of this research, a waiver of informed consent was considered appropriate in Article 458 of the Dutch Medical Treatment Contracts Act (Wet Geneeskundige Behandelingsovereenkomst). Due to the nature of the study, no patient compensation was provided.

Data obtained from the NHR were provided to the research team in pseudonymized form using unique patient identifiers. The 2 keys linking these identifiers to individual patients were securely maintained at the 2 respective treating hospitals. To enable the linkage of the pseudonymized NHR data with additional clinical information extracted from the hospitals’ electronic health records, authorized members of the research team were granted access to the linkage keys at the participating hospitals. Following data linkage, analyses were performed on the linked, pseudonymized dataset. Access to this dataset was restricted to the study authors responsible for data analysis. All results are presented in aggregated form, and no individual-level information is reported.

## Results

### Baseline Characteristics

A total of 131 patients were included (H1-RPM: 34, H1-UC: 64, H2-UC: 33), with a mean age of 73.4 (SD 11.3) years, and 67.2% (n=88) were male. Patient characteristics at baseline were generally similar across cohorts, except for fewer de novo diagnoses of HF in the H2-UC cohort (*χ*²_2_=9.48, *P*=.01, Cramér *V*=.27) and substantially more recent hospital admissions (in the month prior to inclusion) in the H1-RPM cohort (*χ*²_2_=29.22, *P*<.001, Cramér *V*=.47). Table 1 provides a summary of patient characteristics.

**Table 1.** Baseline characteristics of patients included in the study, stratified by hospital and care pathway (N=131).

Characteristic	Value			*P* value^a^
	H1^b^-RPM^c^ (n=34)	H1-UC^d^ (n=64)	H2^e^-UC (n=33)	
Age (y), mean (SD)	72.0 (13.7)	74.2 (10.4)	73.6 (10.4)	.61
Sex, n (**%**)				.23
Male	20 (58.8)	46 (71.9)	22 (66.7)	
Female	14 (41.2)	18 (28.1)	11 (33.3)	
BMI (kg/m^2^), mean (SD)	27.7 (6.2)	26.6 (4.0)	26.8 (5.4)	.63
NYHA^f^-classification, n (%)				.24
I	5 (14.7)	6 (9.4)	5 (15.2)	
II	15 (44.1)	40 (62.5)	13 (39.4)	
III	14 (41.2)	17 (26.6)	9 (27.3)	
IV	0 (0)	0 (0)	1 (3.0)	
Unknown	0 (0)	1 (1.6)	5 (15.2)	
LVEF^g^ (%), mean (SD)	42.94 (14.0)	38.8 (11.8)	36.2 (12.4)	.11
NTproBNP^h^ (ng/L), mean (SD)	728.3 (910.6)	469.6 (808.9)	760.8 (1507.6)	.37
De novo diagnosis, n (%)				.01
No	7 (20.6)	12 (18.75)	15 (45.5)	
Yes	27 (79.4)	52 (82.3)	17 (51.5)	
Unknown	0 (0)	0 (0)	1 (3.0)	
Hospitalization 1-month prior to inclusion, n (%)	34 (100)	36 (56.3)	13 (39.4)	*<*.001
Comorbidities, n (%)^i^				
Hypertension	17 (50.0)	34 (53.1)	14 (42.4)	.69
CVA^j^	3 (8.8)	8 (12.5)	2 (6.1)	.59
Extracardiac arteriopathy	6 (17.6)	8 (12.5)	4 (12.1)	.76
COPD^k^	6 (17.6)	9 (14.1)	1 (3.0)	.15
OSAS^l^	4 (11.8)	5 (7.8)	3 (9.1)	.82
Diabetes mellitus	11 (32.4)	10 (15.6)	8 (24.2)	.11
Thyroid	1 (2.9)	4 (6.3)	2 (6.1)	.79
Malignancy	2 (5.9)	3 (4.7)	1 (3.0)	.64

a *P* values indicate the statistical significance of differences observed among the 3 groups.

b H1: hospital 1.

c RPM: remote patient management.

d UC: usual care.

e H2: hospital 2.

f NYHA: New York Heart Association.

g LVEF: left ventricular ejection fraction.

h NT-proBNP: N-terminal pro–B-type natriuretic peptide.

i Consider the small number of patients with comorbidities when interpreting differences between cohorts.

j CVA: cerebrovascular accident.

k COPD: chronic obstructive pulmonary disease.

l OSAS: obstructive sleep apnea syndrome.

### Total Consultation Volume and Time

The results regarding time spent on consultations and the number of consultations are summarized in Table 2.

The mean number of consultations per patient was significantly higher in the H1-RPM cohort compared to the H1-UC and H2-UC cohorts (*F*_2,128_=19.27, *P*<.001, η²=.23). Consequently, the mean time spent (in min) per patient was significantly higher in the H1-RPM cohort compared to both H1-UC and H2-UC cohorts (*F*_2,128_=31.63, *P*<.001, η²=.34).

**Table 2.** Number of cardiac consultations and consultation time per patient, stratified by hospital and care pathway (N=131)^a^.

Variables	Value			*P* value^b^
	H1^c^-RPM^d^ (n=34), mean (SD)	H1-UC^e^ (n=64), mean (SD)	H2^f^-UC (n=33), mean (SD)	
Number of consultations				
Sum per patient (n)	18.5 (9.3)	9.3 (6.0)	11.6 (6.3)	<.001^g^
HF^h^ nurse consultations (n)	15.3 (8.4)	5.3 (2.4)	8.6 (6.1)	<.001^i^
Phone consultations	2.2 (1.9)	3.2 (2.5)	6.7 (5.6)	<.001^j^
In-person consultations	1.6 (0.9)	1.9 (1.6)	2.0 (1.3)	.45
RPM consultations	11.6 (8.3)	—^k^	—	—
Cardiologist consultations (n)	3.2 (1.7)	4.0 (5.6)	2.9 (1.5)	.43
Phone consultations	1.7 (1.7)	2.1 (5.6)	0.9 (0.9)	.35
In-person consultations	1.4 (1.1)	1.9 (1.2)	2.0 (1.1)	.06
Time spent on consultations				
Sum per patient (min)	266.2 (100.7)	158.9 (51.0)	143.8 (69.7)	<.001^g^
HF nurse consultations (min)	233.2 (95.2)	121.2 (47.9)	113.0 (66.0)	<.001^g^
Phone consultations	43.7 (43.3)	68.1 (48.7)	66.1 (54.8)	.06
In-person consultations	48.1 (31.3)	53.1 (32.7)	47.0 (26.6)	.58
RPM consultations	141.5 (92.1)	—	—	—
Cardiologist consultations (min)	32.9 (17.8)	37.7 (24.3)	30.8 (17.9)	.27
Phone consultations	17.4 (16.1)	17.5 (20.3)	7.0 (7.5)	.01^j^
In-person consultations	15.6 (12.4)	20.2 (13.4)	23.8 (15.5)	.05

a Number of consultations and consultation time are divided by caregiver role and type of consultation.

b *P* values indicate the statistical significance of differences observed among the 3 groups.

c H1: hospital 1.

d RPM: remote patient management.

e UC: usual care.

f H2: hospital 2.

g H1-RPM differs significantly from H1-UC and H2-UC, but H1-UC and H2-UC do not differ significantly from each other, as found in the post hoc test.

h HF: heart failure.

i H1-RPM, H1-UC, and H2-UC all differ significantly from each other, as found in the post hoc test.

j H2-UC differs significantly from H1-RPM and H1-UC, but H1-RPM and H1-UC do not differ significantly from each other, as found in the post hoc test.

k Not applicable.

### Caregiver Consultation Volume and Time

The distribution of care volume and time was different between the care pathways. The patients receiving RPM had significantly more consultations with a nurse practitioner specialized in HF care than patients receiving UC (*F*_2,128_=36.63, *P*<.001, η²=.36), leading to a corresponding significant increase in total consultation time with nurse practitioners specialized in HF care (*F*_2,128_=36.61, *P*<.001, η²=.36). In H2-UC, patients received significantly more phone calls from the nurse practitioner specialized in HF care compared to H1-RPM and H1-UC (*F*_2,128_=15.68, *P*<.001, η²=.20) with a corresponding, albeit nonsignificant, trend in total phone consultation time with the nurse practitioner specialized in HF care (*F*_2,128_=2.96, *P*=.06, η²=.04). No significant difference was found in the number of phone calls between H1-RPM and H1-UC. There was also no significant difference observed for the number and time of in-person consultations with nurse practitioner specialized in HF care (*F*_2,128_=0.80, *P*=.45, η²=0.01; *F*_2,128_=0.55, *P*=.58, η²=0.01, respectively; see Table 2).

Patients receiving RPM care (H1-RPM) had nearly monthly phone contact with a nurse practitioner specialized in HF care, averaging 11.6 (SD 8.3) consultations and a total of 141.5 (SD 92.1) minutes in the first year.

The total number of consultations with a cardiologist did not significantly differ between care pathways (*F*_2,128_=0.85, *P*=.43, η²=.01). There were no significant differences in the number of phone or in-person consultations (*F*_2,128_=1.05, *P*=.35, η²=.02; *F*_2,128_=2.90, *P*=.06, η²=.04, respectively). However, cardiologists in H2-UC spent significantly less time on phone consultations than those in H1-RPM and H1-UC (*F*_2,128_=4.78, *P*=.01, η²=.07). No significant difference was observed in time spent on in-person consultations (*F*_2,128_=3.03, *P*=.05, η²=.05).

### Hospitalizations

The distribution of HF-related hospitalizations per patient did not significantly differ between the 3 cohorts (*χ*²_6_=5.639, *P*=.47, Cramér *V*=.15), and similarly, the average duration of a hospitalization did not differ between the 3 cohorts (*F*_2,57_=2.08, *P*=.16, η²=.07; Table 3).

**Table 3.** Frequency and duration of heart failure (HF)–related hospitalizations per patient, stratified by hospital and care pathway (N=131).

Variables	Value			*P* value^a^
	H1^b^-RPM^c^ (n=34)	H1-UC^d^ (n=64)	H2^e^-UC (n=33)	
Number of patients with, n (%)				.47
0 hospitalization	15 (44.1)	39 (60.9)	16 (48.5)	
1 hospitalization	12 (35.3)	12 (18.8)	7 (21.2)	
2 hospitalization	5 (14.7)	7 (10.9)	6 (18.2)	
3+ hospitalizations	2 (5.9)	6 (9.4)	4 (12.1)	
Mean duration hospitalizations per patient (d), mean (SD)^f^	1.4 (4.0)	2.5 (4.0)	5.5 (9.9)	.16

a *P* values indicate the statistical significance of differences observed among the 3 groups.

b H1: hospital 1.

c RPM: remote patient management.

d UC: usual care.

e H2: hospital 2.

f Only patients with at least one hospitalization are included in this analysis.

## Discussion

### Principal Findings

This study assessed differences in cardiac care delivery and workload distribution associated with RPM implementation compared to UC in a real-world, regional setting in patients with HF. Contrary to common assumptions, the results failed to show an advantage of this implementation of RPM over UC in workload and patient outcomes. Nurses experienced an increased workload, both in terms of number and duration of consultations, which was not compensated for by a reduction elsewhere.

By enriching the numerical data with context from practice, this study offers valuable insights with regard to the implementation of RPM and highlights the value of real-world evaluation when implementing innovative care pathways. Within the context of the regional care network, these results could further allow for more efficient implementation and reimplementation.

### Workload and Shifts in Care Responsibilities

RPM implementation was associated with an increase in the workload of nurse practitioners specialized in HF care, with a higher number and longer duration of consultations, which was not offset by a reduction elsewhere. While we expected a larger shift from UC phone consultations toward RPM consultations, only a slight, nonsignificant shift was observed in patients using RPM. This may partly be explained by the higher proportion of patients with a recent HF hospitalization in the H1-RPM cohort, who are likely to require more intensive follow-up and support. However, our findings are consistent with Ekola et al [23] and Auton et al [24], both of whom also reported increases in nurses’ workload in mixed method studies. Nurses noted that while RPM could ease aspects of their work, it also added time for onboarding, follow-up, and managing false alarms. Given the already high existing workloads, strategies such as better integration of RPM into routine care, task redistribution to new health care roles, improved alarm management, and clinical decision support should be considered to mitigate the additional burden.

This real-world evaluation found no significant differences in hospitalizations between the RPM cohort and the 2 UC cohorts during the first year. In contrast, recent meta-analyses by de Lathauwer et al [20] and Scholte et al [25] both reported a 22% reduction in the first HF-related rehospitalizations. However, most RCTs in these meta-analyses lacked the statistical power to show significant effects on their own and only after pooling significant results emerged. Our study presents a pragmatic, early-stage implementation of RPM, differing from more controlled and larger-scale (though still underpowered) RCTs. This resulted in a limited sample size that reduced statistical power and a potentially less stable H1-RPM cohort, as these patients had more recent hospitalizations, which may have contributed to differences in outcomes. Nevertheless, the data from this study suggest that, with this implementation, the reduction in hospitalizations may not fully offset the increased care time required.

RPM was implemented not only to improve patient health outcomes but also to alleviate the burden on health care professionals. Although agreements were made to reduce cardiologist consultations from every 3 months to every 6 months following RPM implementation, our study found no significant difference in consultation time between the UC and RPM groups. This finding suggests that the RPM system had achieved only limited integration into overall care processes. Unlike scientifically protocolized interventions, RPM was implemented pragmatically, following a standardized care protocol that permitted deviations by clinicians based on patient needs. Such an approach can result in RPM being layered on top of existing pathways, rather than replacing parts of them. While this is a logical and perhaps necessary step in real-world adoption to maintain workability, this approach may have limited RPM’s efficiency. The findings of Bhatia and Maddox [26], which emphasize the need to embed RPM into existing workflows and standardize alert responses for effective integration, are therefore crucial considerations for optimizing this implementation.

### Organizational Improvements

The amount of time spent on RPM consultations was largely due to the patient’s onboarding process, with the first RPM consultation ranging from 60 to 120 minutes. Nurse practitioners specialized in HF care conducted the onboarding at patients’ homes, adding travel time on top of the explanation of RPM. While onboarding for RPM at home was implemented to provide valuable insights into patients’ living conditions and to allow for tailored instructions for technology use, this approach requires a substantial time investment, making it not sustainable for broader implementation. Hailu et al [27] also reported on time-consuming onboarding, especially in patients with lower digital literacy, who often perceived RPM as difficult, resulting in reluctance to adopt RPM. Shifting RPM onboarding to the hospital setting (eg, during hospitalization) offers several advantages: reduced travel time, reinforced importance of RPM, and potentially enhanced patient engagement. This approach aligns with the current practice at H1 (ie, after completion of this study), implemented based on the experiences of nurse practitioners specialized in HF care. The resulting insights were shared within the care network and incorporated into the design of new RPM protocols at other hospitals, where patients are enrolled in RPM pathways either during initial hospitalization for ADHF or at a subsequent outpatient visit.

The RPM implementation in this study followed a decentralized model, with dedicated nurse practitioners specialized in HF care digitally managing patients with HF within their own department, thus expanding their responsibilities. An alternative approach is centralizing RPM, with monitoring duties coordinated by e-nurses, supporting nurse practitioners specialized in HF care and optimizing workflows. In general, e-nurses need a lower level of clinical specialization and are specifically trained to manage patient data and respond to alerts across multiple care pathways and disciplines. This could be particularly beneficial for stable patients with HF who require less specialized care but still contribute to the workload. Centralization would allow nurse practitioners specialized in HF care to focus on more complex cases, with e-nurses serving as a first filter for alerts [28]. However, this shift would require a reallocation of roles, supported by proper training, workflows, and communication pathways to ensure success [29,30]. This model has already been adopted in both H1 and H2 as part of their recently implemented RPM programs. In addition, both hospitals are currently piloting a regional centralized approach, in which stable patients with HF from H2 are monitored by the e-nurses at H1, exhibiting a trust in a more centralized approach from both hospitals.

### Technological Improvements

To reduce the burden of RPM on health care professionals and improve clinical outcomes, improvements in technology are needed besides organizational changes. The types of measurements performed by the patient in this implementation were limited in predicting changes in cardiac filling pressures of the heart, although such changes are strong predictors of eventual HF decompensation. Currently, noninvasive sensors and algorithms capable of detecting these changes are being evaluated in clinical settings and show promise in reducing HF-related hospitalizations, decreasing alert frequency, and extending alert-to-event time, making them strong candidates for future use in RPM [31]. Additionally, the application used in this implementation only collected spot measurements of the symptoms and vital signs, instead of monitoring these continuously. Continuous monitoring could potentially improve the predictive value of these metrics. To support this, AI could be used as a more advanced method for generating alerts, beyond the current threshold-based system, which could reduce the burden on e-nurses [32].

In addition to improving the data collection and processing part of RPM, there is considerable potential to support patients in enhancing their overall quality of life. For example, in the current implementation, patients did not receive feedback from the web application based on the submitted measurements. Previous research has shown that providing patients with educational content, such as links to informational websites or personalized messages based on health data, improves patient outcomes and supports healthier, more informed lifestyles [20,33,34]. These features could also be integrated into existing cardiac rehabilitation programs, which play a crucial role in improving quality of life, exercise capacity, and reducing rehospitalizations in chronic HF [35]. However, adherence is often low due to factors such as logistical challenges, psychological issues, and a high disability burden [36]. Facilitated by RPM, delivering cardiac rehabilitation at home may help overcome these barriers and improve accessibility and adherence [37]. Finally, the Medly Titrate study demonstrated that RPM facilitates faster up-titration to OMT by enabling more frequent patient contact and reducing in-person visits, without increasing adverse events [38]. Integrating RPM into the OMT up-titration process could further improve patient outcomes.

### Further Implementation Within the Regional Network

Beginning in 2022, plans were made for RPM to be implemented across all 4 hospitals affiliated with the regional care network. In collaboration with physicians and nurses within the HF subnetwork, regional guidelines were developed, and consensus was reached on the overall care pathway. These guidelines promote coherence across hospitals by standardizing aspects such as monitoring frequency, monitored parameters, and response protocols. Hospitals retained autonomy in specific implementation decisions, including the choice of RPM platform and the designation of first responders to alerts, depending on local resources and organizational structures. The collaborative structure of the NHN facilitated frequent discussions among caregivers, where challenges and best practices were shared. In line with the plan-do-check-act cycle [39] employed within the care network, further real-world analyses will be conducted to evaluate the impact of this implementation and to identify opportunities for further improvement of the RPM program.

### Limitations

While both participating hospitals used comparable HF care protocols, significant differences were observed between the UC groups from H1 and H2. These between-hospital variations suggest potential differences in UC delivery, which could have affected the interpretation of differences between the UC and RPM groups, for example, in the number and time of consultations. The time of consultations could also have been affected by differences in recording practices of the 2 participating hospitals, with the estimated durations of different consultation types differing slightly.

Although a real-world analysis provides valuable insights into the practical effects of RPM, it is also associated with limitations. For example, unlike RCTs, which can ensure comparable baseline populations, our analysis showed differences between cohorts. RPM selection was based on perceived clinical need, prioritizing more unstable patients who had all experienced an HF-related hospitalization within the month prior to referral. Given the high-risk period following an episode of acute decompensated HF, these patients with recent HF hospitalizations may inherently have higher needs for care and a corresponding need for hospitalizations and consultations, influencing both our outcomes. To assess these potential effects of these population differences, we performed a sensitivity analysis, rerunning our analysis with only the patients with a recent hospitalization (n=83). This sensitivity analysis showed a new significant difference (*F*_2,80_=3.18, *P*=.047, η²=.07), where H1-RPM had a lower number of in-person cardiologist consultations than H2-UC. Furthermore, time spent on phone consultations by cardiologists no longer differs significantly between the 3 groups (*F*_2,80_=2.54, *P*=.09, η²=.06). The full outcomes of this analysis are available in Multimedia Appendix 1.

An additional source of selection bias may have arisen from the patient selection process for RPM. Eligibility was determined by the treating cardiologist and nurse practitioner based not only on clinical need but also on the patients’ perceived ability to successfully engage with remote monitoring. As a result, patients with greater perceived digital literacy, cognitive functioning, and self-management skills may have been preferentially enrolled. This may have introduced differences between groups that were not captured in the baseline characteristics and could have influenced outcomes.

Moreover, the relatively small sample size of 131 patients limited the statistical sensitivity of the study. A power calculation using G*Power version 3.1.9.7 (Heinrich-Heine-Universität Düsseldorf) [40] indicated that, with 3 groups and a total sample size of 131 participants, the study had 80% power at α=.05 to detect moderate-to-large effects of η²≥0.07 (Cohen *f*=0.27). Consequently, smaller but potentially clinically relevant effects may have remained undetected. Nonetheless, the real-world dataset offers important insights into RPM’s implementation in clinical practice, highlighting challenges and barriers that highly protocolized RCTs may not capture. Analyzing these data allows for better understanding and directions for the optimization of RPM to enhance patient outcomes and health care efficiency.

### Conclusion

This study, examining a real-world, pragmatic implementation of RPM, showed that implementation was associated with increased time spent and a higher number of consultations for nurse practitioners specialized in HF care, while no clear reduction was observed in time spent or the number of consultations for cardiologists. While the study had limited statistical sensitivity to detect small effects, no clear differences were observed between the RPM cohort and the UC cohorts in the number or duration of rehospitalizations. As improvements in clinical outcomes have already been shown in RCTs, it is paramount that these outcomes are also realized in real-life clinical practice, through proper guidelines and effective strategies for implementing RPM. Based on reflections on this real-world retrospective dataset, potential areas for improvement in future RPM implementation can be identified at both organizational and technological levels.

## Supplementary material

10.2196/89187 Multimedia Appendix 1 Number of cardiac consultations and consultation time per patient, stratified by hospital and care pathway.

10.2196/89187 Checklist 1 STROBE checklist for cohort studies.

## Abbreviations

H1: hospital 1
H2: hospital 2
HF: heart failure
NHN: Netherlands Heart Network
NHR: Netherlands Heart Registration
OMT: optimal medical therapy
RCT: randomized controlled trial
RPM: Remote Patient Management
STROBE: Strengthening the Reporting of Observational Studies in Epidemiology
UC: usual care
