# Supplementary material for: Real-World Impact of Remote Patient Management on Care Consumption and Care Time in Heart Failure: Retrospective Cohort Study
Source: JMIR Form Res. 2026 Sep 3;10:e89187. doi: 10.2196/89187 (PMC13540973; doi:10.2196/89187)
Supplement: Supplementary file 1 [file formative-v10-e89187-s001.docx]

# Supplementary Table A: sensitivity analysis

Supplementary Table A: Number of cardiac consultations and consultation time per patient, stratified by hospital and care pathway. Number of consultations and consultation time are divided by care giver role and type of consultation. P-values in the final column indicate the statistical significance of differences observed among the three groups. Sensitivity analysis including only patients with a hospitalization at most 1 month prior to inclusion.

| **Number of consultations** | | | | | |
| --- | --- | --- | --- | --- | --- |
| **Variables** | | **Values** | | | ***P*-value** |
|  |  | **H1-RPM (n=34)** | **H1-UC**  **(n=36)** | **H2-UC**  **(n=13)** |  |
| Sum Per Patient (n), mean (SD) | | 18*.*5 (9*.*3)^A^ | 9*.*7 (7.5)^B^ | 10*.2* (3.3)^B^ | **<0.001** |
| **HF-nurse Consultations (n), mean (SD)** | | 15*.*3 (8*.*4)^A^ | 4.9 (2.4)^B^ | 6.8 (3.7)^C^ | **<0.001** |
|  | Phone Consultations | 2*.*2 (1*.*9)^A^ | 3.4 (2.6)^B^ | 5.0 (3.3)^C^ | **0.002** |
|  | In-person Consultations | 1.6 (0.9) | 1.6 (0.9) | 1.9 (1.2) | 0.48 |
|  | RPM Consultations | 11.6 (8.3) | ------ | ------ | ------ |
| **Cardiologist Consultations (n), mean (SD)** | | 3.2 (1.7) | 4.7 (7.2) | 3.3 (2.0) | 0.41 |
|  | Phone Consultations | 1.7(1.7) | 2.8(7.4) | 0.9 (0.9) | 0.44 |
|  | In-Person consultations | 1.4 (1.1)^A^ | 1.9 (1.2)^AB^ | 2.4 (1.4)^B^ | **0.047** |

| **Time spent on consultations** | | | | | |
| --- | --- | --- | --- | --- | --- |
| **Variables** | | **Values** | | | ***P*-value** |
|  |  | **H1-RPM (n=34)** | **H1-UC**  **(n=36)** | **H2-UC**  **(n=13)** |  |
| Sum Per Patient (minutes), mean (SD) | | 266.2 (100.7)^A^ | 150.7 (47.8)^B^ | 124.2 (44.1)^B^ | **<0.001** |
| **HF-nurse Consultations (minutes), mean (SD)** | | 233*.*2 (95*.*2)^A^ | 114.2 (45.6)^B^ | 94.6 (44.1)^B^ | **<0.001** |
|  | Phone Consultations | 43.7 (43.3) | 66.9 (50.1) | 48.5 (33.6) | 0*.*09 |
|  | In-person Consultations | 48.1 (31.3) | 47.2 (21.5) | 46.2 (26.6) | 0*.*97 |
|  | RPM Consultations | 141.5 (92.1) | ------ | ------ | ------ |
| **Cardiologist Consultations (minutes), mean (SD)** | | 32.9 (17.8) | 36.5 (21.4) | 29.6 (16.0) | 0.50 |
|  | Phone Consultations | 17*.*4 (16*.*1) | 16.7 (15.9) | 6.9(5.6) | 0.09 |
|  | In-Person consultations | 15.6 (12.4) | 19.9 (14.1) | 22.7 (13.0) | 0.20 |
